# Preliminary Investigation Towards a Safety Tool for Swine Brucellosis Diagnosis by a Proteomic Approach Within the One-Health Framework

**DOI:** 10.3390/ijms26041517

**Published:** 2025-02-11

**Authors:** Simona Sagona, Fabrizio Bertelloni, Barbara Turchi, Paola Roncada, Elena Tafi, Filippo Fratini, Antonio Felicioli, Domenico Cerri

**Affiliations:** 1 Department of Veterinary Science, Pisa University, viale delle Piagge 2, 56124 Pisa, Italy; fabrizio.bertelloni@unipi.it (F.B.); barbara.turchi@unipi.it (B.T.); filippo.fratini@unipi.it (F.F.); antonio.felicioli@unipi.it (A.F.); domenico.cerri@unipi.it (D.C.); 2 Department of Pharmacy, Pisa University, via Bonanno 6, 56126 Pisa, Italy; 3 Department of Health Science, University “Magna Graecia” of Catanzaro, viale Europa, 88100 Catanzaro, Italy; roncada@unicz.it; 4 CREA Research Centre for Agriculture and Environment, via di Corticella 133, 40128 Bologna, Italy; elenat93@libero.it

**Keywords:** brucellosis, electrophoresis, infectious disease, serology, western blot, Brucellergene

## Abstract

Brucellosis is a zoonosis that affects domestic and wild animals, causing reproductive disorders and significant economic losses in livestock. *Brucella melitensis*, *B. abortus*, and *B. suis* are the main agents of brucellosis in livestock and humans, thereby their control and eradication are crucial. Serological tests based on identification of antibodies against *Brucella* smooth lipopolysaccharides (sLPS) in the serum of infected animals are traditionally used. This approach shows two main limits: (i) tests can give false positives due to the similarity of *Brucella* sLPS with the LPS of other Gram-negative bacteria; (ii) antigen production represents a possible risk of zoonoses. In this work, a proteomic approach, starting from *B. melitensis* Brucellergene, was employed to identify possible *Brucella* antigenic proteins useful for a more specific and safe serological diagnosis. Four proteins binding to the infected swine serum were identified: (i) “probable sugar-binding periplasmic protein *B. abortus* str 2308A”; (ii) “peptide ABC transporter substrate-binding protein *B. melitensis*”; (iii) “GntR family transcriptional regulator *B. melitensis*”; (iv) “conserved hypothetical protein *B. melitensis* M28”. These proteins could be promising specific antigens for serological investigations in swine. In the near future, these antigenic proteins could be synthesized in vitro and used to produce a safer and more specific diagnostic kit.

## 1. Introduction

*Brucella* spp. are Gram-negative coccobacillus bacteria that cause diseases in various animal species, including humans [1]. In domestic animals, the disease occurs as a chronic infection which results in placentitis and abortion in pregnant females, and orchitis and epididymitis in males, causing significant economic losses in livestock farms [1,2]. *Brucella* spp. can persist and replicate within the phagocytic cells of the reticuloendothelial system and in non-phagocytic cells such as trophoblasts [1]. When the vacuoles containing *Brucella*-individuals are fused with lysosome for the bacteria degradation, the lysosomal proteins are excluded, and the *Brucella*-containing vacuoles are associated with the endoplasmic reticulum which represents the intracellular replication site for *Brucella* [1].

Among the twelve known *Brucella* species, the most frequent agents of brucellosis in livestock and humans are *Brucella melitensis*, *Brucella abortus*, and *Brucella suis* [3]. Several biovars of these *Brucella* species exist, and it is possible to distinguish five biovars of *B. suis* [4]. Although *B. melitensis* and *B. abortus* can be transmitted to pigs because of contact with ruminants, swine brucellosis is mainly caused by *B. suis*, biovars 1, 2, and 3 [4,5]. *B. suis* bv. 1 and 3 are rarely reported in Europe while *B. suis* bv. 2 is largely diffused in East Europe. It was also introduced in Italy, where it was detected in domestic pigs and wild boars [6,7,8]. However, in Italy, Bertelloni and colleagues [9] reported that swine brucellosis seems to have a very limited spread in intensive farms. *B. suis* bv. 2 recognizes as principal hosts swine and hares, but it has been also detected in cows, causing seroconversion to traditional tests for bovine brucellosis, without clinical signs [10,11]. Human infections by *B. suis* bv. 2 were rarely reported [12].

Traditional methods for the diagnosis of brucellosis include bacteria isolation and characterization from biological samples, and serological tests. In addition, several molecular methods including PCR, PCR-restriction fragment length polymorphism (RFLP), and Southern blot, allowed, to a certain extent, the differentiation of *Brucella* species and some of their biovars [13]. Serological methods are often employed in control and eradication programs to initially identify the possible positive animals. These methods are based on the detection of antibodies against the lipopolysaccharides (sLPS) of smooth *Brucella* strains generated by infected animals [8,14]. The monoclonal antibodies against A and M antigens recognize the smooth LPS of *B. suis* strains; however, the first does not recognize *B. melitensis* strains and second does not bind to *B. abortus* strains [8]. The Rose Bengal test (RBT), complement fixation test (CFT), indirect/competitive enzyme-linked immunosorbent assay (I/C ELISA), and fluorescence polarization assay (FPA) are the validated serological tests commonly used for swine brucellosis diagnosis [15]. The serologic tests used to diagnose brucellosis were mostly developed for the detection of the A dominant, *B. abortus* O side-chain in infected cattle; consequently, these diagnostic tests have lower sensitivity and specificity when applied in swine than in cattle [8]. In general, serological tests present some limitations, mainly concerning specificity and sensitivity, especially when screening individual animals [15,16]. For these reasons test interpretation is generally conducted at a group or herd level, requiring bacterial isolation or molecular assays to confirm serologic data [8].

Other Gram-negative bacteria, namely *Escherichia coli* O157:H7, *Vibrio cholerae* O1, *Salmonella* group N (O:30), and *Yersinia enterocolitica* O:9, can induce the production of antibodies that cross-react with the *Brucella* sLPS antigens [17,18,19]. Particularly, *Y. enterocolitica* is widespread in swine populations and has an O-antigen LPS chain nearly identical to that of *Brucella*, resulting in a significant number of false positive serological reactions [14,15,20]. The RBT is used as a screening test, but it lacks specificity for discriminating reactions caused by smooth *Brucella* from other bacteria cross-reactions [21]. The CFT is generally used as a confirmatory test, but it has a reduced sensitivity for *B. suis* infection diagnosis, and it is affected by cross-reactions with other bacteria [21]. The FPA resulted in a very good performance test but in chronically infected animals reported a low sensitivity, as well as in other serological tests [21].To overcome these shortcomings, the development of alternative immunoblotting methods is being investigated to increase the specificity and sensitivity of serological tests for brucellosis diagnosis on a rough strain of *Brucella melitensis* (88/131) [22]; on outer membrane proteins (OMPs) of the Rev 1 strain of *B. melitensis* [23]; and on an extract of *B. abortus* and *B. melitensis* [24]. In all these techniques, the authors had to cultivate the bacteria, exposing operators to the risk of infection since *Brucella* can easily infect the operator by airborne transmission [25].

Among the new techniques tested, one includes the use of Brucellergene OCB (Rhône-Mérieux, Lyon, France) which is a commercial antigen produced from *B. melitensis* B115, previously employed in swine for in vitro serological tests, such as ELISA [14], and for in vivo skin tests [26], showing significant specificity and the ability to discriminate false positive serological reactions. Brucellergene OCB is a mixture of more than 20 cytoplasmic proteins including T-cell antigens, *Brucella* bacterioferritin, and P39 proteins prepared from a rough (deficient in smooth LPS) mutant of *Brucella melitensis B115* [27]. Bertelloni and colleagues [28] used Brucellergene as a tool to detect brucellosis-affected animals by Dot Blot, confirming its validity and ease of use in swine brucellosis serological diagnosis. Although the use of Brucellergene is risk-free for operators, this technique also requires the presence of *Brucella* in the laboratory, exposing operators to the risk of infection.

This work aims to identify *Brucella* antigenic proteins in Brucellergene as a starting point for the development of safer immunological techniques for *Brucella* screening.

## 2. Results

**Figure 1:**
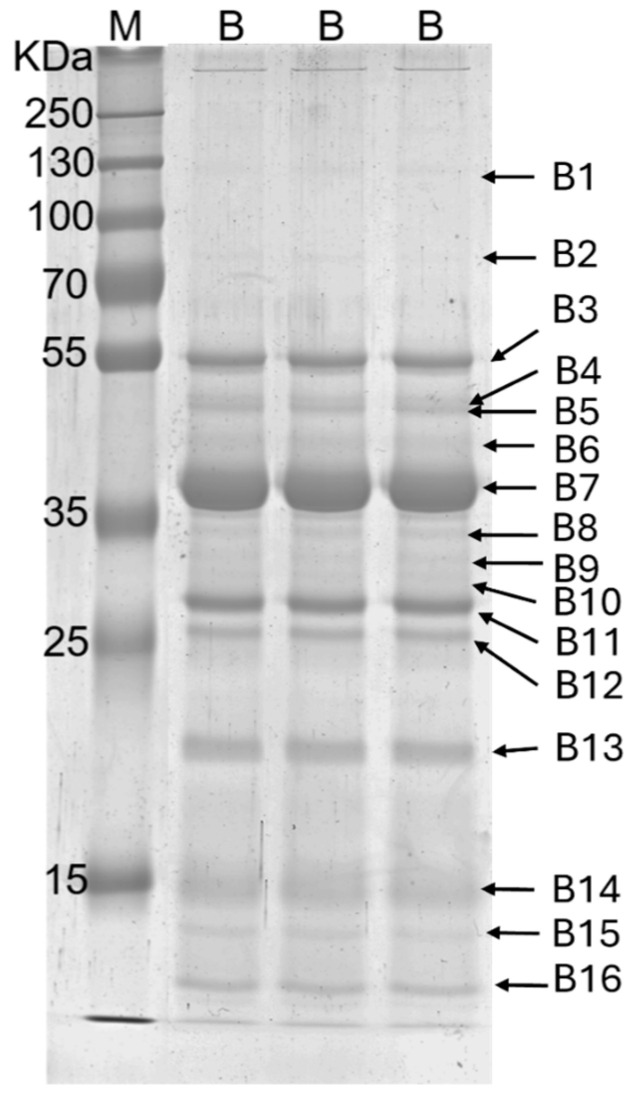
SDS-PAGE 7.5% T, 2.6% C. B: Brucellergene OCB (Rhône-Mérieux, France) an antigen produced from *Brucella melitensis* rough strain B115 employed in swine for in vitro serological tests; M: markers (15–250 kDa); B1–B16 main protein bands, detected in the electrophoretic gel whose molecular weights have been calculated.

Figure 1 reports an SDS-PAGE of a Brucellergene OCB sample. Sixteen protein bands with a molecular weight of 115 (B1), 84 (B2), 55 (B3), 50 (B4), 48 (B5), 44 (B6), 39 (B7), 34 (B8), 33 (B9), 31 (B10), 29 (B11), 27 (B12), 20 (B13), 13 (B14), 12 (B15), and 11 (B16) kDa were observed.

**Figure 2:**
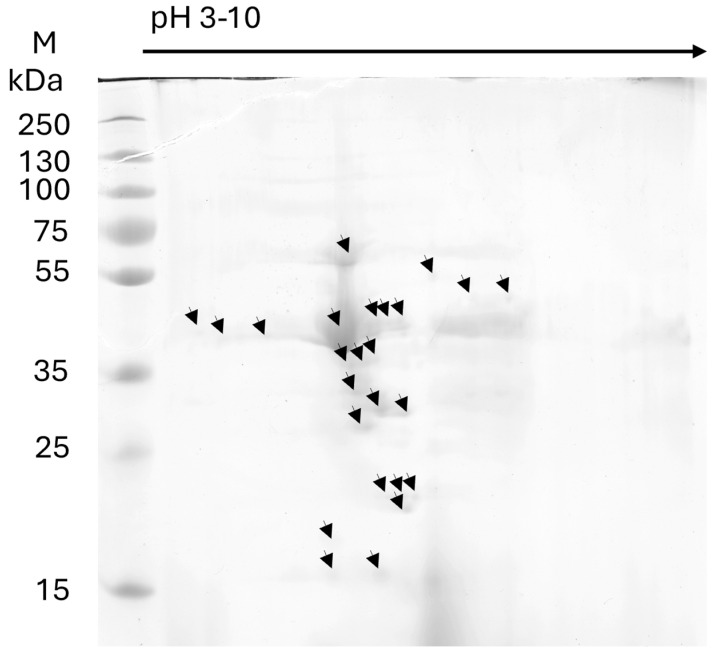
2D electrophoresis of Brucellergene OCB (Rhône-Mérieux, France) an antigen produced from *Brucella melitensis* rough strain B115 employed in swine for in vitro serological tests. M = markers (15–250 kDa). Strip 7 cm pH 3–10 linear; 7.5% T, 2.6% C. The arrows indicate the main spots detected by the 2D SDS-PAGE.

In addition to the SDS-PAGE of the Brucellergene OCB sample, 2D electrophoresis was also performed (Figure 2). In 2D electrophoresis, at least 20 spots were detected with molecular weights corresponding to those obtained in the bands of the SDS-PAGE, while the isoelectric point ranged between pH 4.8 and 7.8.

**Figure 3:**
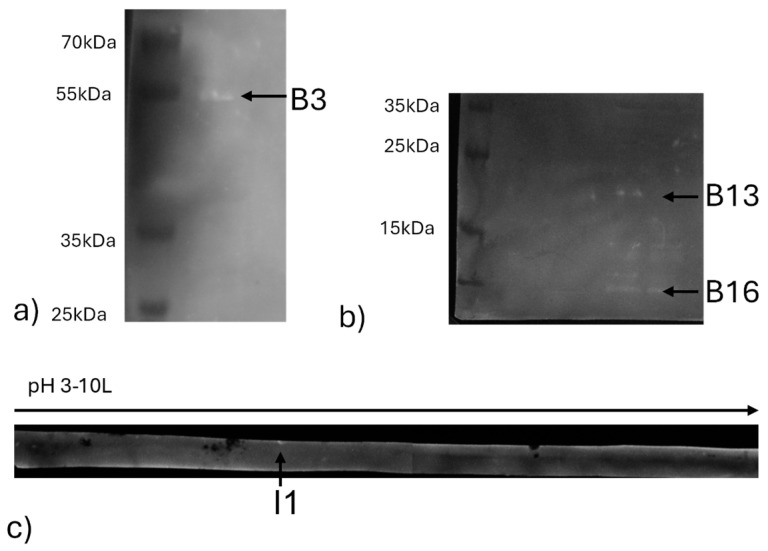
Western Blot analysis of proteins of Brucellergene separated by SDS-PAGE (**a**,**b**) or by isoelectric focusing electrophoresis (**c**); B3, B13, B16, and I1 indicated the bands that gave a luminol-detected signal due to the binding with positive anti-*Brucella* swine serum, and were subjected to cutting for identification by mass spectrometry.

**Figure 4:**
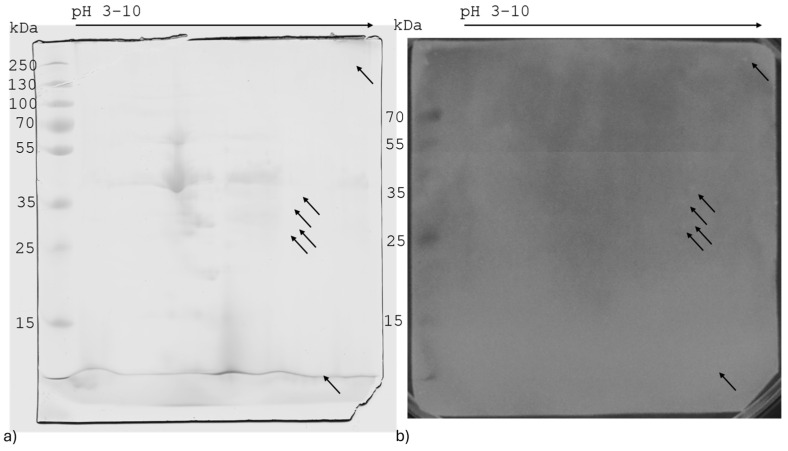
2D SDS-PAGE T 7.5%, C 2.6%, and Western Blot analysis Brucellergene proteins. The arrows indicate the main spots that were detected by Western Blot (**b**) and that were not present in the 2D SDS-PAGE (**a**).

The results of the Western Blot applied to SDS-PAGE are reported in Figure 3. In Figure 3a,b, three bands which correspond to B3, B13, and B16 and with 55, 20, and 11 kDa of molecular weight, respectively, were able to bind positive anti-*Brucella* swine serum. The Western Blot of the 2D gel of Brucellergene on nitrocellulose did not show spots corresponding to the electrophoretic gel (Figure 4 and Supplementary Figure S1). The strip resulting from isoelectrofocusing was directly blotted, showing a band named I1 at an isoelectric point around pH 5.5–6 (Figure 3c).

Proteins identified by mass spectrometry are shown in Table 1.

## 3. Discussion

The use of Brucellergene OCB by Dot Blot as a tool to detect brucellosis-affected animals has already been investigated by Bertelloni and colleagues [28]. Bertelloni and colleagues [9] tested 374 swine sera for brucellosis using the Rose Bengal Test (RBT), complement fixation test (CFT), and Dot Blot, using Brucellergene as an antigen. To verify the concordance of Dot Blot using CFT as the gold standard, they observed a concordance value of at least 91% [9]. *Y. enterocolitica* is mainly responsible for cross-reactions in swine [8]. The Dot Blot, using Brucellergene as an antigen and an anti-*Yersinia enterocolitica* serum as an antibody, did not show cross-reaction, suggesting a promising specificity [28]. Since the Brucellergene bound the anti-*Brucella* swine serum but did not bind the anti-*Yersinia* serum, we investigated by Western Blot which proteins within those contained in Brucellergene could bind *Brucella*-positive swine serum. It was assumed that these proteins were not able to cross-react with the anti-*Yersinia* serum as well as the whole of Brucellergene.

The *Brucella* proteome has already been studied by several authors [24,29,30,31,32]. Hamidi and colleagues [33] developed a ribosomal proteome-based mapping for the establishment of biomarker profile libraries to identify *B. abortus* and *B. melitensis*, as well as elucidating refined differences between virulent and vaccine sstrains.

To the best of our knowledge, Brucellergene had never been investigated using a proteomic approach. In agreement with several authors who have previously investigated the *Brucella* proteome, the present results highlighted *B. melitensis* proteins with molecular weights in the range of 10–116 kDa [29,30,34].

Regarding the 2D SDS-PAGE, no matching spots to bind to *Brucella*-sera were found by the Western Blot approach. This is because chemiluminescent detection is more sensitive and produces signal at lower protein concentrations than the staining of the gel with coomassie G250, according to the product sheet provided by the company [35]. It can be speculated that a milder treatment of the Brucellergene might preserve the proteins in their native forms and thus might improve the resolution of the Western Blot. Further investigations are therefore needed to clarify this aspect.

Among the detected bands, only those which reacted with the *Brucella*-serum in the Western Blot were identified by Mass Spectrometry. These bands corresponded to four proteins identified as follows: a probable sugar-binding protein, a peptide ABC transporter substrate-binding protein, a GntR family transcriptional regulator, and a conserved hypothetical protein.

A class of sugar-binding proteins with molecular weight and isoelectric points corresponding to the protein found by this investigation (probable sugar-binding periplasmic protein *B. abortus* str 2308A) has been also observed overexpressed in the proteome of Rev1 (an attenuated strain of *B. melitensis*) [36]. Rev 1 is considered a highly effective vaccine in the control of brucellosis in small ruminants in many countries [36]. A sugar-binding protein with a similar IP and molecular weight has been also detected in both Rev 1 and in a *B. melitensis* virulent strain, 16M [29,37]. To the best of our knowledge, this protein in terms of amino acid composition is not like any cloned *Yersinia enterocolitica* protein (% homology 0%).

The second protein identified belongs to ATP-binding cassette (ABC) transporters, a large group of membrane protein complexes that couple the transport of a substrate across the membrane to the hydrolysis of ATP [38]. In prokaryotes, ABC transporters are localized to the plasma membrane, and ATP is hydrolyzed on the cytoplasmic side [38]. Furthermore, ABC transporters are characterized by two nucleotide-binding domains (NBDs) and two transmembrane domains (TMDs) [39]. An ABC transporter acts as a transporter of different molecules across biological membranes and participates in a variety of biological processes, such as maintaining osmotic pressure balance inside and outside the cell, antigen presentation, cell differentiation, and bacterial immunity [40]. A protein at about 60 kDa binding all sera was also found by Wareth and colleagues [24], applying a Western Blot to an extract of *B. abortus* and *B. melitensis* using cattle, buffaloes, sheep, and goat sera as primary antibodies. This protein could correspond to the molecular weight of the protein identified in this investigation as the peptide ABC transporter substrate-binding protein. When comparing the amino acid sequence of the probable sugar-binding protein with other proteins, a percentage of 99.52% homology with the peptide ABC transporter substrate-binding protein was observed. It could be speculated that *Brucella*-positive swine serum might bind to these two proteins in a similar portion of the amino acid sequences. Comparing the amino acid sequence of peptide ABC transporter substrate-binding protein with other proteins, a percentage of homology higher than 86% was only observed with proteins belonging to the genus *Brucella*, thus suggesting that this is a genus-specific protein. The peptide ABC transporter substrate-binding protein is similar in terms of amino acid composition to cloned *Yersinia enterocolitica* proteins, with a percentage of homology of lower than 41%.

The third protein identified is a GntR regulator, an important virulence factor in *Brucella* playing important roles in the maintenance of fatty acid concentrations, amino acid catabolism, organic acids production, the regulation of carbon catabolism and the degradation of complex organics [41]. Furthermore, some research indicates that GntR mutants show reduced virulence [40,42].

The fourth protein identified is a conserved hypothetical protein. Wagner and colleagues [43] had already identified in the *B. melitensis* proteome several hypothetical low-molecular-weight proteins whose function, to the best of our knowledge, is undefined. Comparing the amino acid sequence of the GntR family transcriptional regulator or conserved hypothetical protein with other proteins, a percentage of homology higher than 82% or 70%, respectively, was only observed with proteins belonging to the genus *Brucella*, thus suggesting that they are genus-specific proteins. The GntR family transcriptional regulator protein is similar in terms of amino acid composition to *Yersinia enterocolitica* proteins with a percentage of homology lower than 49% while the conserved hypothetical protein is not like any *Yersinia enterocolitica* protein cloned.

Concerning the subcellular localization prediction, both probable sugar-binding protein and ABC transporters belong to periplasmic protein [44]. Brucellae can reversibly modify their cell envelope to adapt to changes in the host intracellular microenvironment and improve their survival by modifying the host immune response [24]. Zai and colleagues [31] investigated the resistance of *B. abortus* to various stresses (e.g., antibacterial stress, nutrient starvation stress, and physicochemical stress) and observed that some proteins, including the ABC transporter ones, were still produced by the bacterium despite the stressful conditions. As they are also expressed under stress conditions for the bacterium, they could be a target for antibodies produced by the infected animal. It could therefore be speculated that ABC transporter proteins may be a target for a probable *Brucella* identification test.

Some authors have investigated the proteomes of some *Brucella* species and virulent/attenuated strains to search for species-specific proteins as a basis for new diagnostic screening methods [29,30,34,44]. Eschenbrenner and colleagues [30] investigated the *B. melitensis* vs. *B. abortus* proteomes and observed the presence of ABC transporter proteins in both species. This could suggest that ABC transporter proteins are not species-specific proteins and therefore could be further investigated as possible antigens to produce a general immunological kit for the identification of *Brucella* infections.

The “probable sugar-binding periplasmic protein *B. abortus* str 2308A”, “peptide ABC transporter substrate-binding protein *B. melitensis*”, “GntR family transcriptional regulator *B. melitensis*”, and “conserved hypothetical protein *B. melitensis* M28” identified in this work could be produced in vitro, providing the basis for the development of a diagnostic kit to avoid *Brucella* culture required for large-scale antigen production. In vitro synthesis of the protein could be performed in a molecular biology laboratory by drawing ad hoc primers and cloning the protein via a vector into cell cultures (e.g., *Escherichia coli*). After purification by SDS-PAGE and specific columns, the protein could be tested by Dot Blot with anti-*Brucella*-positive swine serum.

## 4. Materials and Methods

### 4.1. Material

Since Bertelloni and colleagues [28] reported that only positive serum had a cross-reaction with Brucellergene, only positive serum, from a free-ranged farm of “cinta senese” pigs, in South Tuscany (Siena province, Italy), was used in this investigation. Employed serum was stocked at −20 °C until processed. The antigen was the Brucellergene OCB (Rhône-Mérieux, France), produced from *B. melitensis* rough strain B115, provided by “Istituto Zooprofilattico della Lombardia e dell’Emilia Romagna Bruno Ubertini, Brescia, Italy” and by “Istituto Zooprofilattico Sperimentale dell’Abruzzo e del Molise G. Caporale, Teramo, Italy” for Western Blot (WB). 

### 4.2. Sodium Dodecyl Sulphate—PolyAcrylamide Gel Electrophoresis (SDS-PAGE)

The Brucellergene total protein content was measured by Qubit 2.0 Fluorometer (Invitrogen, Waltham, MA, USA). Ten µg of total protein of Brucellergene was loaded into 7.5% T, 2.6% C separating polyacrylamide gels (1.5 mm thick). A 10–250 kDa pre-stained protein Sharpmass^TM^ V plus protein MW marker (Euroclone, Pero, Italy) was also carried out. SDS-PAGE was performed at 20 mA/gel and at 15 °C using SE 260 mini vertical electrophoresis (GE Healthcare, Chicago, IL, UK).

### 4.3. 2DE (Two-Dimensional Gel Electrophoresis) SDS-PAGE

Isoelectric focusing electrophoresis was performed at 20 °C on an IPGphor III apparatus (GE Healthcare) following a previously reported protocol [45,46]. The volumes of protein extract corresponding to 75–150 μg of total proteins were mixed with a rehydration solution (Urea 7 M, Thiourea 2 M, Chaps 2%, dithiothreitol 0.5%, IPG 1%, and a trace of Bromophenol blue) and loaded on 7 cm (pH 3–10) strips, rehydration time 9 h, 50 mA/strip. For some strips, the Western Blot method was applied.

Prior to SDS-PAGE, the IPG strips were equilibrated for 8 min in 50 mM Tris-HCl pH 8.8, 30% Glycerol, 6 M Urea, 4% SDS, 2% dithiothreitol and afterwards for 12 min in 50 mM Tris-HCl pH 6.8, 30% Glycerol, 6M Urea, 4% SDS, 2.5% Iodoacetoamide and bromophenol blue. The following SDS-PAGE was performed using self-cast 7.5% T, 2.6% C separating polyacrylamide gels according to Laemmli [47], without stacking gel.

### 4.4. Western Blot (WB)

Considering an SDS-PAGE where two gels run, proteins were fixed in one of the gels by 40% methanol and 10% acetic acid solution for 30 min. Gel was stained in Coomassie brilliant G colloidal solution [48], discolored with water, scanned by an Epson Perfection V750 Pro (Suwa, Nagano, Japan), and elaborated by ImageJ software, version 1.54 [49]. For the second gel, proteins were transferred to a nitrocellulose membrane (0.45 µm size, Thermo Scientific, Waltham, MA, USA) by ECL TE 70 PWR Semi-dry transfer unit (GE Healthcare), 0.8 mA/cm^2^, for 4 h and 30 min. Western blot was performed according to Iovinella et al. [50], with modifications. The membrane was exposed to serum samples at 1:200 concentrations, with 30 min of incubation time in a dark place and with inactivation at 58 °C ± 2 °C for 60 min. Afterwards, the membrane was incubated for 1 h at RT with a polyclonal rabbit anti-Pig IgG-(H+L) antibody, HRP conjugated (Bethyl Laboratories, Montgomery, TX, USA) diluted 1:10,000. The reaction was detected by ClarityTM Western ECL substrate Kit (Biorad Laboratories, Hercules, CA, USA). Twenty seconds of exposure to a Nikon D5100 camera (Tokyo, Japan) fitted with a 50 mm f/1.4 lens and 12 mm extension tube, detected the chemiluminescent signal in a dark room [51].

### 4.5. Mass Spectrometry

The bands corresponding to those that reacted with the antibody in the Western Blot were selected into the gel, excised and sent to a Mass Spectrometry Center (CISM, Florence University, Florence, Italy) where mass spectrometry was applied, and proteins were identified.

The excised bands were destained and proteins digested as reported by Dani et al. [52]. Each peptide mixture was submitted to capillary-LC-μESI-MS/MS analysis on an Ultimate 3000 HPLC (Dionex, San Donato Milanese, Milan, Italy) coupled to a LTQ Orbitrap mass spectrometer (Thermo Fisher, Bremen, Germany). Peptides were concentrated on a precolumn cartridge PepMap100 C18 (300 μm id × 5 mm, 5 μm, 100 Å, LC Packings Dionex, Sunnyvale, CA, USA) and then eluted on a homemade capillary column packed with Aeris Peptide XB-C18 phase (180 μm id × 15 cm, 3.6 μm, 100 Å, Phenomenex, Torrance, CA, USA) at 1 μL/min. The loading mobile phases were as follows: 0.1% TFA in H_2_O (phase A) and 0.1% TFA in CH_3_CN (phase B). The elution mobile phases composition was H_2_O 0.1% formic acid/CH_3_CN 97/3 (phase A) and CH_3_CN 0.1% formic acid/ H_2_O 97/3 (phase B). The elution program was as follows: 0 min, 4% B; 10 min, 40% B; 30 min, 65% B; 35 min, 65% B; 36 min, 90% B; 40 min, 90% B; 41 min, 4% B; 60 min, 4% B. Mass spectra were acquired in positive ion mode, setting the spray voltage at 1.8 kV and the capillary voltage and temperature at 45 V and 200 °C, respectively, and the tube lens at 130 V. Data were acquired in data-dependent mode with dynamic exclusion enabled (repeat count 2, repeat duration 15 s, exclusion duration 30 s); survey the MS scans that were recorded in the Orbitrap analyzer in the mass range 300–2000 *m*/*z* at a 15,000 nominal resolution at *m*/*z* = 400; then up to three of the most intense ions in each full MS scan were fragmented (isolation width 3 *m*/*z*, normalized collision energy 30) and analyzed in the IT analyzer. Monocharged ions did not trigger MS/MS experiments. The acquired data were searched with Mascot 2.4 search engine (Matrix Science Ltd., London, UK) against *Brucella* protein sequences downloaded from NCBI.

## 5. Conclusions

Four proteins able to bind *Brucella*-positive swine serum were identified (a probable sugar-binding protein, a peptide ABC transporter substrate-binding protein, a GntR family transcriptional regulator, and a conserved hypothetical protein) by proteomic and Western Blot approaches. All of them could be exploited to enhance the specificity of serological investigations. Among these proteins, the peptide ABC transporter substrate seems the most promising one to be used as a specific antigen because *Brucella* can produce it even under stress conditions. Although Brucellergene is safe to handle, standardized, and already potentially useful for the serological investigation of *Brucella* by Dot Blot, it requires, however, the cultivation of *Brucella* in the laboratory. As future steps for serological assays in swine brucellosis, the most suitable antigenic proteins could be synthesized in vitro, avoiding the cultivation of the Brucellae and thus reducing the risk of infection for operators by airborne transmission.

Further investigation will be then needed to test these proteins and verify whether they can provide a safety tool for serological diagnosis in screening diagnostic for swine brucellosis, breeding screening or monitoring plans.

## Figures and Tables

**Table 1:** Proteins identified across 7.5% T, 2.6% C polyacrylamide gels from Brucellergene OCB (Rhône-Mérieux, France) produced from *B. melitensis* rough strain B115, the only one which bonded with *Brucella*-positive swine serum. For each protein, scores resulting from protein sequence alignment in BLAST (total score, E-value, and % identity), molecular weight (MW), isoelectric point (IP), number of amino acids, and % identity with *Yersinia enterocolitica* proteins are reported.

Band	I1	B3	B13	B16
Accession number	EEP62043.1	WP_118874259.1	WP_087907809.1	ADZ65660.1
Protein description	Probable sugar-binding periplasmic protein *Brucella abortus* str 2308A	Peptide ABC transporter substrate-binding protein *Brucella melitensis*	GntR family transcriptional regulator *Brucella melitensis*	Conserved hypothetical protein *Brucella melitensis* M28
Total score	858	1083	470	207
E-value	0.0	0.0	3.00 × 10^−170^	4.00 × 10^−70^
% identity	100%	92.62%	99.57%	100%
MW (kDa)	45.3	57.39	26.7	12.28
I.P.	5.0	4.99	5.5	10.62
aa	428	525	233	102
% identity with *Yersinia enterocolitica* proteins	0%	<41%	<49%	0%

## Data Availability

The raw data supporting the conclusions of this article will be made available by the authors upon request.

## Supplementary Materials

The following supporting information can be downloaded at: https://www.mdpi.com/article/10.3390/ijms26041517/s1.
