# Supplementary material for: Preliminary Investigation Towards a Safety Tool for Swine Brucellosis Diagnosis by a Proteomic Approach Within the One-Health Framework
Source: Int J Mol Sci. 2025 Feb 11;26(4):1517. doi: 10.3390/ijms26041517 (PMC11855111; doi:10.3390/ijms26041517)
Supplement: Supplementary file 1 [file ijms-26-01517-s001.zip › ijms-3425349-supplementary.pdf]

Supplementary materials to: Preliminary investigation towards a safety tool for swine brucellosis diagnosis by a proteomic approach within the one-health framework

Simona Sagona[1,2,†*], Fabrizio Bertelloni[1†], Barbara Turchi[1], Paola Roncada[3], Elena Tafi[4], Filippo Fratini[1], Antonio Felicioli[1], Domenico Cerri[1]

[1]Department of Veterinary Science, Pisa University, viale delle piagge 2, 56124, Pisa, Italy

[2]Department of Pharmacy, Pisa University, via Bonanno 6, 56126, Pisa, Italy

[3]Department of Health Science, University "Magna Graecia" of Catanzaro, viale Europa, 88100, Catanzaro, Italy

[4]CREA Research Centre for Agriculture and Environment, Via di Corticella 133, 40128 Bologna, Italy

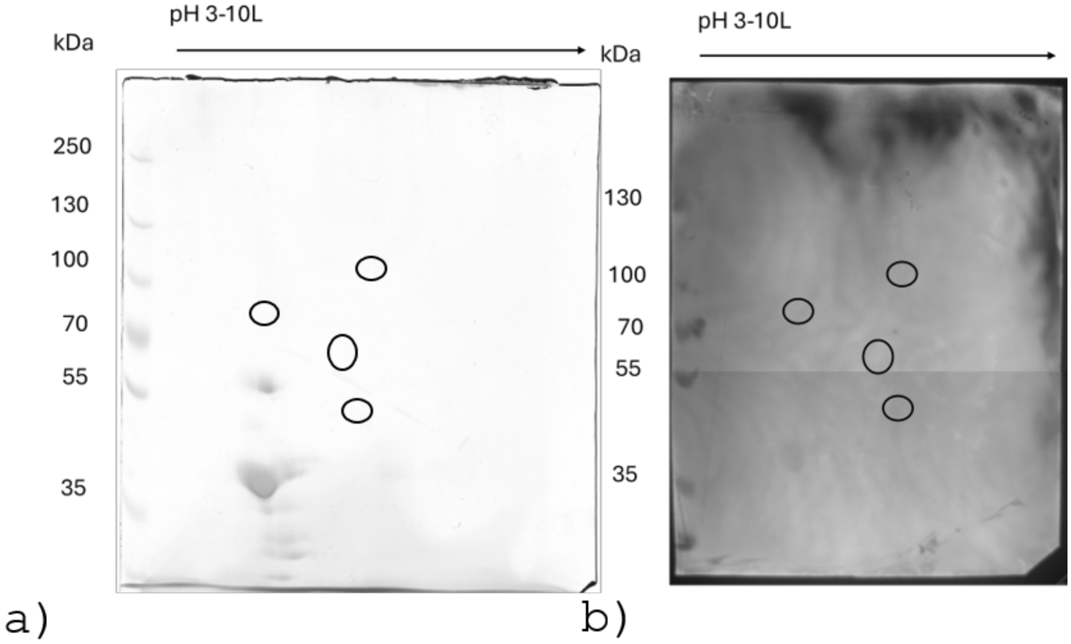

Figure S1. 2D SDS PAGE T7.5% C2.6% and Western Blot analysis of proteins of Brucellergene. The circles indicate the main spots detected by Western Blot (**b**) that were not present in the 2D SDS PAGE (**a**).
